# Editorial: Thrombotic Disorders, Prothrombotic Abnormalities and COVID-19

**DOI:** 10.3389/fmed.2021.676137

**Published:** 2021-04-15

**Authors:** Pierpaolo Di Micco, Manuel J. Núnez Fernández, Alexandra Bura Riviere, Benjamin Brenner

**Affiliations:** ^1^Department of Internal Medicine, Fatebenefratelli Hospital of Naples, Naples, Italy; ^2^Department of Internal Medicine, Complejo Hospitalario Universitario de Pontevedra, Pontevedra, Spain; ^3^Department of Vascular Medicine, CHU Toulouse, Toulouse, France; ^4^Thrombosis and Hemostasis Unit, Rambam Health Care Campus, Haifa, Israel

**Keywords:** COVID-19, thrombosis, pulmonary embolism, immune thrombocytopenia, stroke, metformin, d-dimer, deep vein thrombosis

Hypercoagulability is one of the major hallmarks of the COVID-19 course, manifesting as venous and arterial thromboembolism along with cardiovascular and lung failure. While biomarkers, such as a persistent increase in D-dimer and troponin levels, have been suggested to reflect disease severity, improved risk assessment is vital for the optimal anticoagulant management of this life-threatening condition.

In this collection, authors from different countries confirm that the prothrombotic conditions characterized by increased d-dimer and troponin are present in inpatients with COVID-19 (Goudot et al. and Tassiopoulos et al.) and this increase may also influence the approach to thromboprophylaxis in this clinical setting. Regarding venous thromboembolism prevention inpatients next to orthopedic surgery, in fact, no differences with traditional thrombphylactic doses of low molecular weight heparin approach has been found in a study (Perazzo et al.), while inpatients hospitalized for COVID-19 in regular clinic ward without surgical urgencies or intensive care supports may benefit of increased prophylactic doses of enoxaparin or regular doses of fondaparinux (Russo et al.).

Yet, in the daily clinical practice, based on extended prophylactic anticoagulation, the best way to detect venous thromboembolism with objective methods remains a matter of debate, and the optimal timing of ultrasound scan or lung CT scan are a part of this problem (Lapébie et al.). Some authors recommend to perform radiological diagnostic evaluation at the disease onset, while others propose such evaluation if d-dimer levels are increasing, considering hospitalization for COVID-19 an additional confounding factor to thrombosis development. A second diagnostic assessment for thrombotic disorders may be useful prior to modification of thomboprophylactic regimen. Clinical overt pulmonary embolism and/or fatal pulmonary embolism may occur, in fact, not only in critically ill patients (Benito et al.) in ICU but also in patients in a non-intensive ward during the hospitalization (Benito et al. and Gratz et al.).

In addition, the occurrence of atherothrombotic diseases is not infrequent in COVID-19 (Sattar et al.). Cardiovascular risk factors such as hypertension, obesity, and diabetes may trigger arterial thrombotic events in this clinical setting in the presence of prothrombotic gene polymorphism associated with atherthrombosis, like ACE insertion/deletion polymorphism (Calabrese et al.). Hence, several drugs have been suggested to modulate the atherothrombotic risk with metformin being one of most efficacious in this setting (Al-Kuraishy et al.).

Autoimmune diseases modifying the clotting balance have been reported as a late complication of COVID-19. From a pathophysiological point of view, the cytokine storm with imbalanced autoimmunity has been identified as a main player in these clinical conditions (Guo et al.). In this setting, also COVID-19-associated immune thrombocytopenia may occur (Hindilerden et al.) and a differential diagnosis with heparin induced thrombocytopenia should be performed because it may further complicate this challenging clinical scenario, with possible aggravation of thrombosis and disseminated intravascular coagulation.

The extreme clinical variability of the novel pandemic disease requires robust advances in diagnostics and management strategies. This is vital, since the COVID-19 pandemic is ongoing, while vaccination is still in its early stages.

## Author Contributions

PD planned the editorial. MN identified references. AR and BB performed revision of text. All authors contributed to the article and approved the submitted version.

## Conflict of Interest

The authors declare that the research was conducted in the absence of any commercial or financial relationships that could be construed as a potential conflict of interest.

